# Supplementary material for: Centripetal nuclear shape fluctuations associate with chromatin condensation in early prophase
Source: Commun Biol. 2023 Jul 12;6:715. doi: 10.1038/s42003-023-05074-9 (PMC10338514; doi:10.1038/s42003-023-05074-9)
Supplement: Supplementary file 2 — Supplementary Information [file 42003_2023_5074_MOESM2_ESM.pdf]

# Supplementary Information

## Centripetal nuclear shape fluctuations associate with chromatin condensation before mitosis

Viola Introini, Gururaj Rao Kidiyoor, Giancarlo Porcella, Pietro Cicuta and Marco Cosentino Lagomarsino*

* Corresponding author: Marco Cosentino Lagomarsino

**Email:** Marco.Cosentino-Lagomarsino@ifom.eu



| | |
|---|---|
| **Tables** | **2-4** |
| **Figures** | **5-16** |
| **Videos** | **17** |

**Table S1**. Fluctuation properties of the nuclear envelope (NE) across the cell cycle

| Cell cycle | RBCs | G1 | S | G2 | EP | LP |
|---|---|---|---|---|---|---|
| Number of cells | 31 | 20 | 15 | 19 | 9 | 11 |
| Effective bending modulus ($10^{-20}$ J) | 12.5 ± 0.8 | 2.3 ± 0.7 | 1.4 ± 0.4 | 2.2 ± 1.2 | 2.2 ± 0.5 | 1.3 ± 0.4 |
| Effective tension ($10^{-7}$ N/m) | 12.0 ± 0.8 | 17.1 ± 2.2 | 23.6 ± 2.4 | 19.5 ± 2.7 | 3.9 ± 0.4 | 1.8 ± 0.5 |
| Radius (μm) | 4.18 ± 0.04 | 7.5 ± 0.1 | 8.5 ± 0.3 | 11.1 ± 0.5 | 9.6 ± 0.3 | 9.2 ± 0.4 |
| Relaxation time mode 3 (s) | 0.035 ± 0.008 | 2.1 ± 0.1 | 2.4 ± 0.1 | 2.0 ± 0.2 (14 cells) | 2.4 ± 0.2 | 2.9 ± 0.2 |

Nuclear radius, effective tension, effective bending modulus and fluctuation timescale of mode 3, measured by flickering spectrometry, were compared at different times during the cell cycle. 6-34 modes were considered for the HeLa cell cycle, modes 8-20 for RBCs. Measurements were consolidated from 3 independent experiments for each phase. The nuclear radius is the mean calculated from the center of the nucleus. The average values and the respective standard errors of the mean (SEM) were calculated from the number of cells indicated in the table. Data shown in **Fig. 1d-g**.

**Table S2.** P values < 0.05 for Figures in the manuscript

| Fig. 1 | | Fig. 2 | | Fig. 3 | |
|---|---|---|---|---|---|
| **Effective tension ($10^{-7}$ N/m)** | **P values** | **Effective tension ($10^{-7}$ N/m)** | **P values** | **Skewness** | **P values** |
| G1 – ME | $2.45 \times 10^{-5}$ | Calyculin A early | 0.0142 | G1-G2 | 0.0282 |
| G1 – ML | $1.95 \times 10^{-5}$ | Calyculin A late | $1.65 \times 10^{-4}$ | G1-ME | $1.03 \times 10^{-4}$ |
| S – G2 | 0.01 | Latrunculin A 20 min post-treatment | $8.7 \times 10^{-5}$ | G1-ML | $6.04 \times 10^{-6}$ |
| S – ME | $3.47 \times 10^{-4}$ | Latrunculin A 50 min post-treatment | $3.0 \times 10^{-4}$ | S-ME | 0.0079 |
| S – ML | $4.75 \times 10^{-8}$ | **Radius (μm)** | **P values** | S-ML | $6.25 \times 10^{-5}$ |
| G2 – ME | $4.79 \times 10^{-5}$ | Calyculin A late | 0.0024 | G2-ML | $2.81 \times 10^{-4}$ |
| G2 – ML | $5.96 \times 10^{-6}$ | | | ME-ML | 0.0079 |
| ME – ML | 0.005 | | | Calyculin A Control -ME | 0.0021 |
| **Radius (μm)** | **P values** | | | Calyculin A Control -ML | $2.31 \times 10^{-5}$ |
| G1 – S | 0.0044 | | | VE822 | 0.0265 |
| G1 – G2 | $1.4 \times 10^{-7}$ | | | | |
| G1 – ME | $2.45 \times 10^{-5}$ | | | | |
| G1 – ML | $2.58 \times 10^{-4}$ | | | | |
| S – G2 | $7.53 \times 10^{-5}$ | | | | |
| S – ME | 0.015 | | | | |
| G2 – ME | 0.04 | | | | |
| G2 – ML | 0.02 | | | | |
| **Relaxation time mode 3 (s)** | **P values** | | | | |
| G1 – ML | 0.0027 | | | | |
| S – ML | 0.0293 | | | | |
| G2 – ML | 0.0031 | | | | |

P values reported for data shown in **Fig. 1,2, and 3**. Pairwise statistical comparisons were performed using the two-sided Mann-Whitney U test and the two-sample t-test after checking whether the data followed a Gaussian distribution.

**Table S3.** Biophysical properties of NE upon biochemical perturbations

| Treatment | Number of cells | Tension ($10^{-7}$ N/m) | Radius (µm) | Relaxation time mode 3 (s) |
|---|---|---|---|---|
| Calyculin A pre-treatment | 21 | 13.3 ± 1.7 | 8.2 ± 0.1 | 2.1 ± 0.2 (5 cells) |
| Calyculin A early | 9 | 7.2 ± 1.1 | 7.9 ± 0.2 | 2.7 ± 0.1 (5 cells) |
| Calyculin A late | 12 | 2.6 ± 0.4 | 6.3 ± 0.3 | |
| Latrunculin A pre-treatment | 13 | 9.9 ± 1.4 | 12.1 ± 0.4 | |
| Latrunculin A 20 min | 11 | 2.9 ± 0.4 | 11.4 ± 0.4 | |
| Latrunculin A 50 min | 9 | 2.2 ± 0.3 | 11.6 ± 0.5 | |
| Trichostatin A (pre -treatment) | 29 | 8.56 ± 1.0 | 9.8 ± 0.1 | |
| Trichostatin A | 36 | 9.7 ± 1.2 | 9.7 ± 0.1 | |

Nuclear radius and tension measured by flickering spectrometry upon Calyculin A and Latrunculin A perturbations. Two biological replicates were measured for each condition, and the same nuclei were recorded before and after biochemical treatments. The relaxation time of mode 3 is reported in the case of Calyculin A treatment. The average values and the respective standard errors of the mean (SEM) were calculated from the number of cells indicated in the table. Data shown in **Fig. 3**.

**Figure S1.** Nuclear (cross-section) area increases throughout the cell cycle in HeLa cells

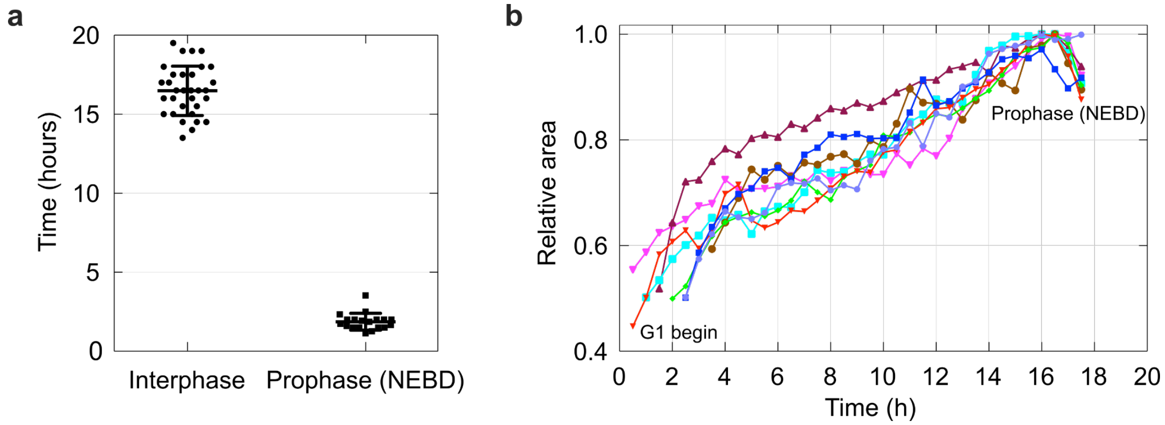


Hela cell nuclei are tracked using the H2B-mCherry marker to determine the cell cycle staging. (**a**) Average time taken by cells to complete one cell cycle, separately in interphase (after formation of daughter nuclei post mitosis) and in prophase at the nuclear envelope breakdown (NEBD, from beginning of condensation to formation of daughter nuclei). Interphase: -16.48 ± 0.2754 h, n=32; prophase (NEBD): -1.75 ± 0.1118 h, n=10. (**b**) Relative growth of nuclear cross section area across cell cycle. Observed a continuous increase in nuclear area throughout the cell cycle reaching maximum at late G2 and with a minor reduction at the onset of prophase and the nuclear envelope breakdown (n=8). This is in agreement with data on nuclear radii in **Fig. 1d**.

**Figure S2.** The radius/tension dynamics of single cells agrees with the average behavior.

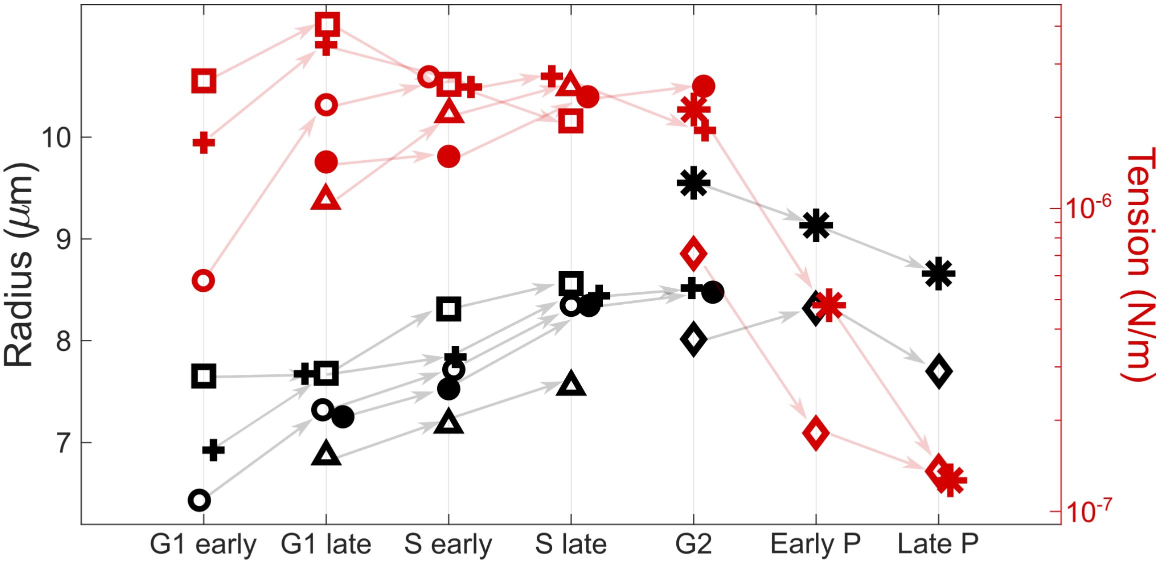


The plot shows nuclear shape properties of 7 cells followed along their cycle starting either from arrest in G1 or late G2, monitoring radius (black) and effective tension (red). Their behavior is generally in agreement with the average behavior shown in **Fig. 1**.

**Figure S3.** Autocorrelation plot of the temporal evolution of mode 3 from the static spectrum

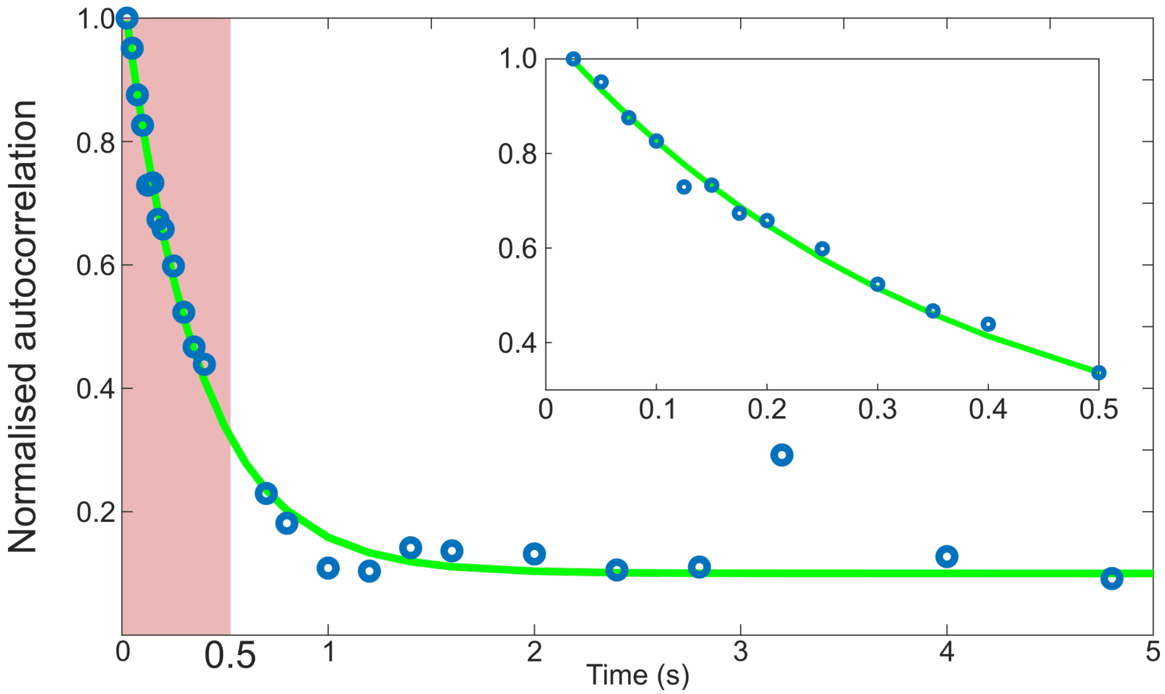


Typical autocorrelation function of the fluctuation amplitudes for mode 3 as function of time. This function has been fitted for times below 0.5 s with a single exponential to obtain the relaxation time of nuclei, as suggested by theory (Yoon *et al*. 2009 [3]).

**Figure S4.** Calyculin A treatment does not affect Lamin A/C phosphorylation

**a**

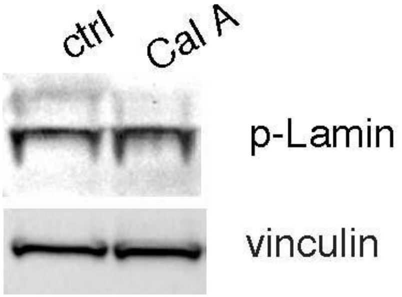


**b**

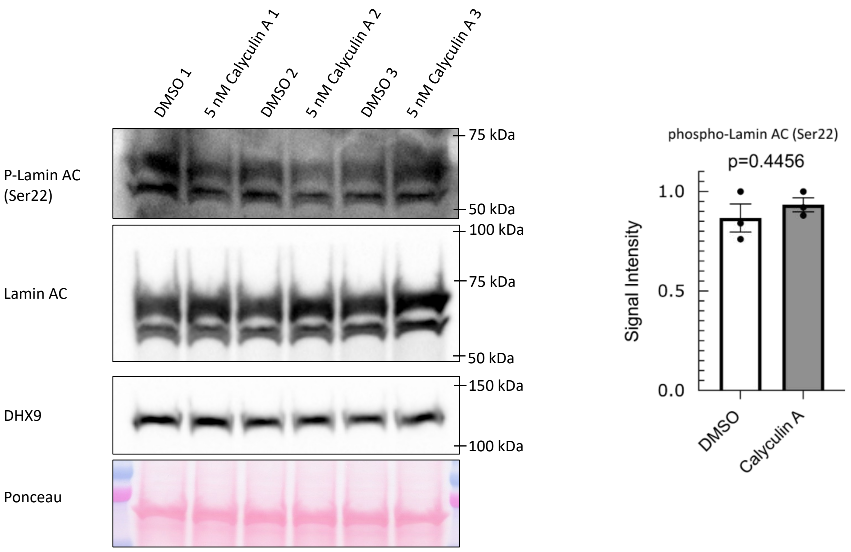


**a**: Western blot analysis of control HeLa cells and HeLa cells treated with 5 nM Calyculin A for 20 minutes, probed for phospho-Lamin A/C (Ser22) and Vinculin (gel representative of 2 copies that confirm the data). Calyculin A treatment does not result in changes in the level of Lamin A/C phosphorylation. **b**: Western blot analysis of 3 independently treated set of samples of exponentially growing HeLa cells treated with 5 nM DMSO or 5 nM Calyculin A for 20 minutes, probed for phospho-Lamin A/C (Ser22), total Lamin A/C and DHX9. Calyculin A treatment does not significatively alter the level of Lamin A/C phosphorylation.

**Figure S5.** Control experiment: actomyosin contractility does not cause nuclear invaginations

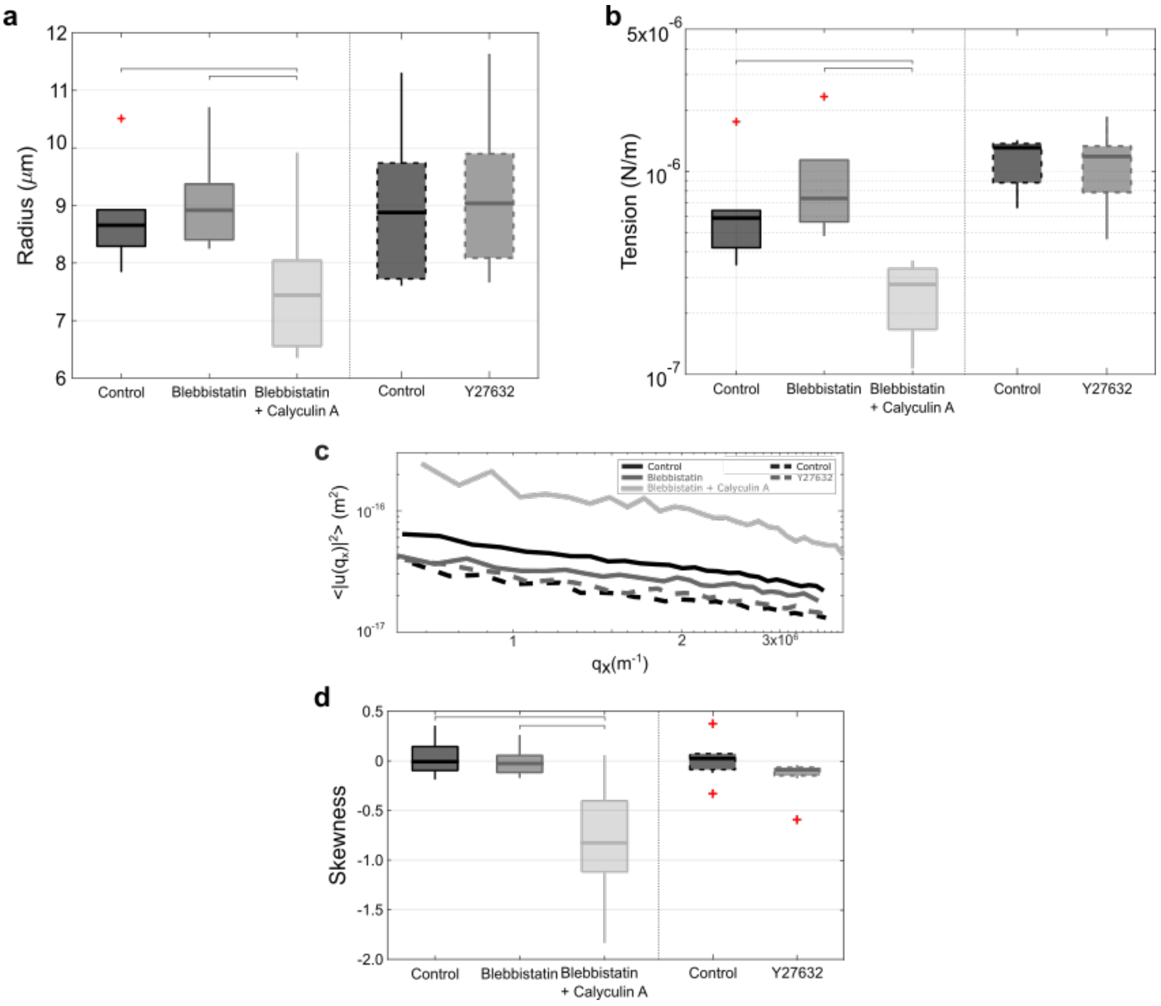


Experiments to rule out the role of myosin-2 mediated contractility as origin of nuclear invaginations due to calyculin A treatment. Blebbistatin and Y27632 (contrary to blebbistatin which is inactivated by blue light, Y27632 is not affected by illumination) confirm that the invagination phenotype is independent of actomyosin contractility. Nuclear radius (**a**), tension (**b**), amplitudes of nuclear envelope fluctuations (**c**) and skewness (**d**) of fluctuation distribution were measured for the same 8 unsynchronised cells before and after blebbistatin and blebbistatin+calyculin A (45 min incubation: 30 min with blebbistatin + 15 min incubation with both), and after Y27632 treatment (30 min incubation). No significant difference for all the properties measured between control and blebbistatin/Y27632 treatments, while the difference is significant with respect to the combination of blebbistatin + calyculin A as expected from **Fig.3**. P values highlighted in the figure: radius 0.0493 (control - blebbistatin + calyculin A) and 0.0295 (blebbistatin - blebbistatin + calyculin A); tension 0.0027 (control - blebbistatin + calyculin A) and 0.0016 (blebbistatin - blebbistatin + calyculin A); skewness 0.0174 (control - blebbistatin + calyculin A) and 0.0035 (blebbistatin - blebbistatin + calyculin A). Pairwise statistical comparisons were performed using the two-sided Mann-Whitney U test and the two-sample t-test after checking whether the data followed a Gaussian distribution.

**Figure S6.** Effect of Trichostatin A on NE

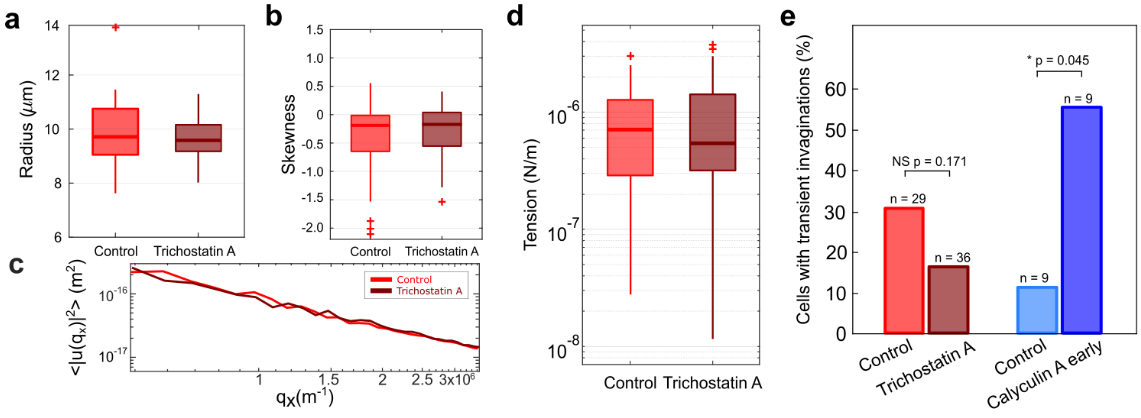


No significant difference can be observed on NE fluctuations after treatment with Trichostatin A (TSA): **a**) nuclear mean radius, **b**) skewness, **c**) NE fluctuation spectra, **d**) NE tension. **e**) Percentage of cells showing transient invaginations of NE for the same time interval halved after Trichostatin A treatment (number of cells considered n=36 with respect to its control n=29 (31% in control, 17% TSA), on the contrary they increase after Calyculin A treatment (n=9) with respect to its control (n=9) (11% control, 55% Calyculin A). Chi-square test was used for statistical pairwise comparison.

**Figure S7.** The shape of invaginations in early prophase is compatible with prediction of a pinning force

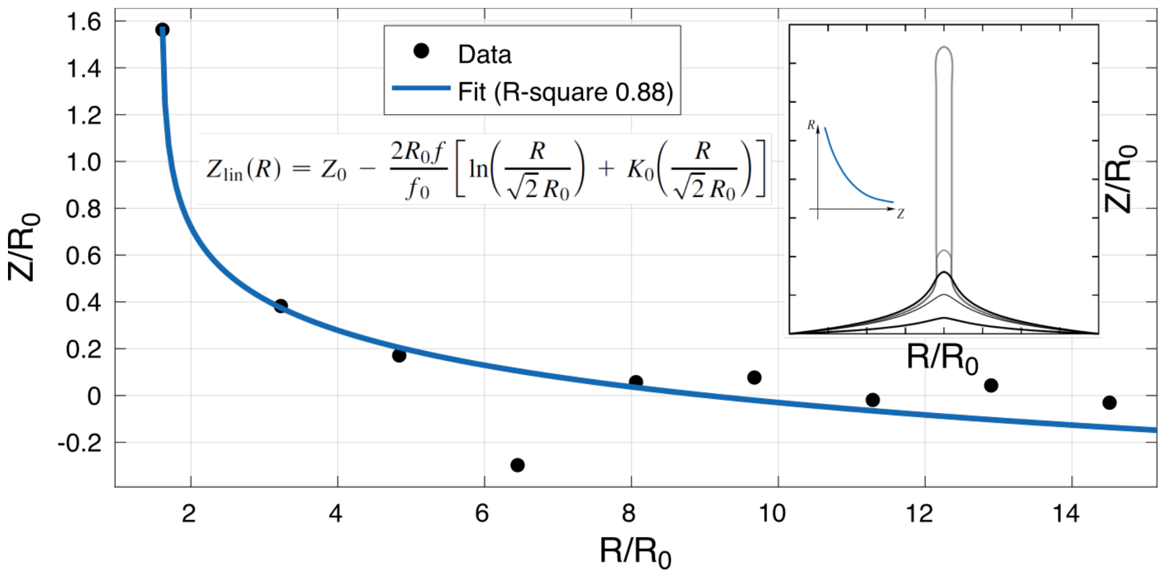


The shape of invaginations at the early stage of nuclear prophase resemble the shape of an emerging tube when the membrane is pulled by a point force f. The data for one side of the invagination are fitted with the equation in the inset formulated by Derényi *et al*. 2002 [29], by knowing $f_0$ and $R_0$, which are related to the bending modulus and tension of the membrane (in our case effective bending modulus and tension).

**Figure S8.** Absence of separation between NE and chromatin globule surface (CGS) at the invagination sites

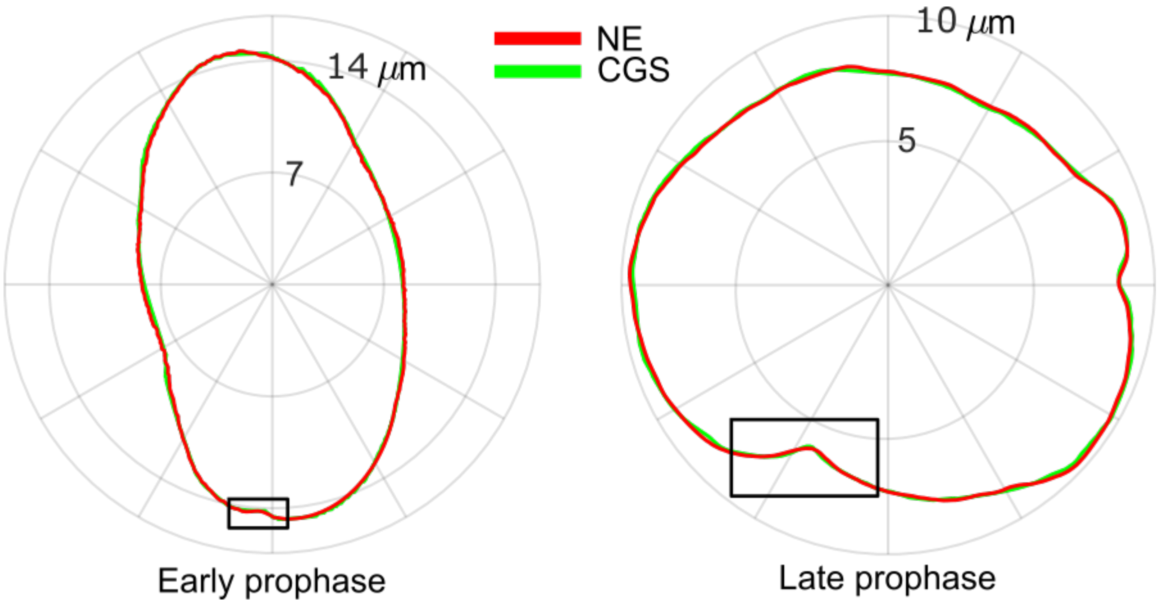


Instantaneous contours of NE and CGS of two representative nuclei from 11 cells in both early and late prophase stage do not exhibit any separation in the site of invaginations (highlighted in the black square). No separation between NE and CGS was reported throughout the invagination period for all cells analysed.

**Figure S9.** Correlation between histones and transient invagination during mitosis

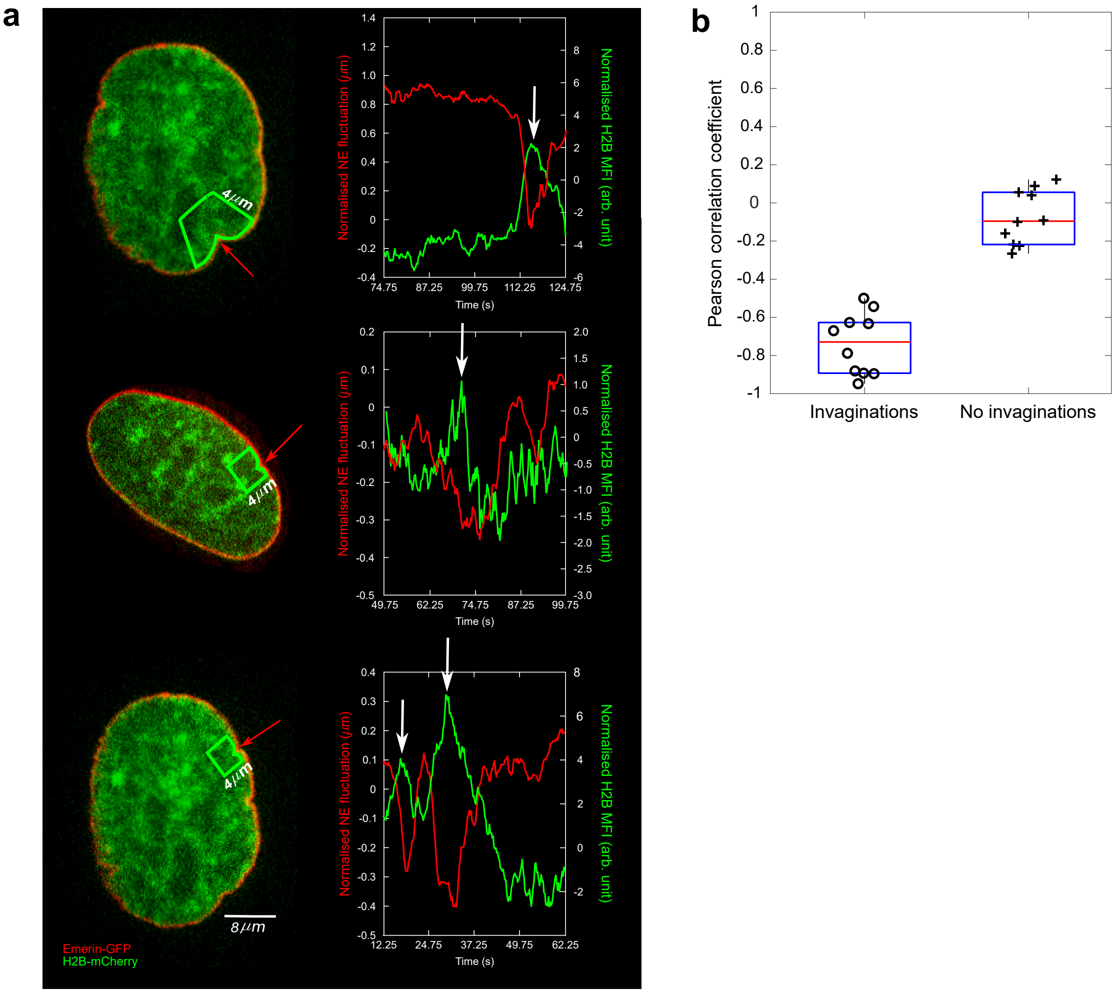


**a**) Examples of the negative correlation between the fluorescence signal of histones (green) in the proximity of the invagination (area highlighted in the green in the image of the nuclei) and the relative NE deformation (red) for nuclei in early and late prophase. The green plots show the mean fluorescence intensity of the area of interest normalized for the mean fluorescence intensity of the entire nuclei during transient invaginations, while the red plots show the nuclear contour for the angle at which the invagination is at its maximum and subtracting the initial frame. As shown in **SI Video 8**, the third nucleus has repetitive invaginations in the same section of the contour (white arrows). **b**) Pearson correlation coefficient between NE deformations and chromatin fluorescence, comparing locations with invaginations and locations with no invaginations for the same total time. The Pearson correlation is close to -1 for invaginations and close to 0 for control regions. 10 invaginations and 10 controls were analyzed in total.

**Figure S10. No cross correlation is found between local transient invaginations and microtubule**

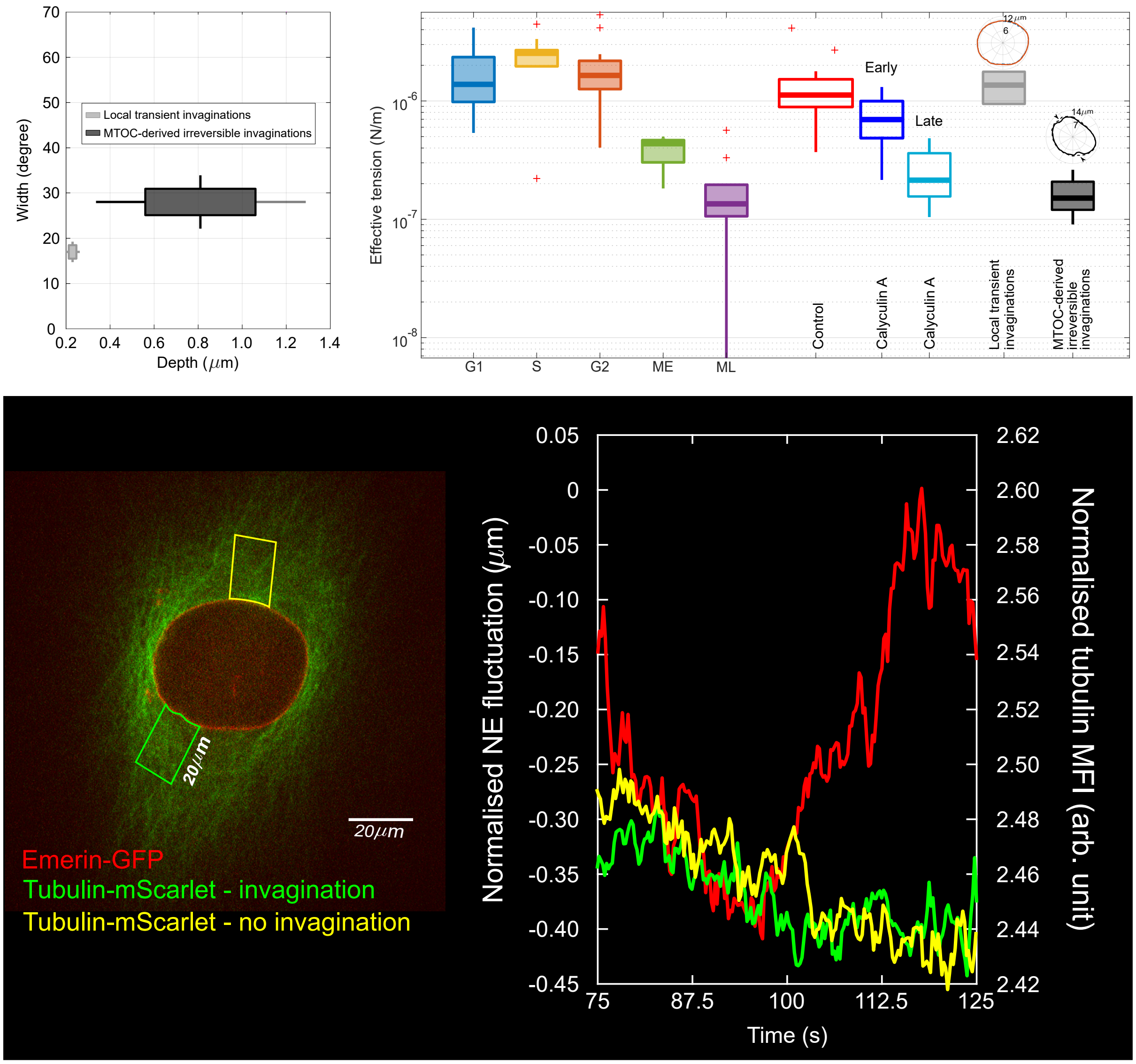


Microtubules do not cause local transient invaginations but MTOCs larger and irreversible ones. a) Scatter plot of values for depth and width of invaginations at their maximum. b) Comparison of effective nuclear tension of cells presenting local invaginations (**SI Video 11**) and MTOC-derived invaginations (**SI Video 12**) with respect to cells at different points of the cell cycle and after Calyculin A treatment. c) Plot of fluorescence signal of microtubules (green) in the proximity of the invagination (area highlighted in the green around the nucleus) and in another area without invaginations (yellow), and the relative NE deformation (red). The green and yellow plots show the mean fluorescence intensity of the area of interest normalized for the mean fluorescence intensity of the tubulin, while the red plots show the nuclear contour for the angle at which the invagination is at its maximum after subtracting the initial frame.

**Figure S11.** Effect of ATR inhibition on NE

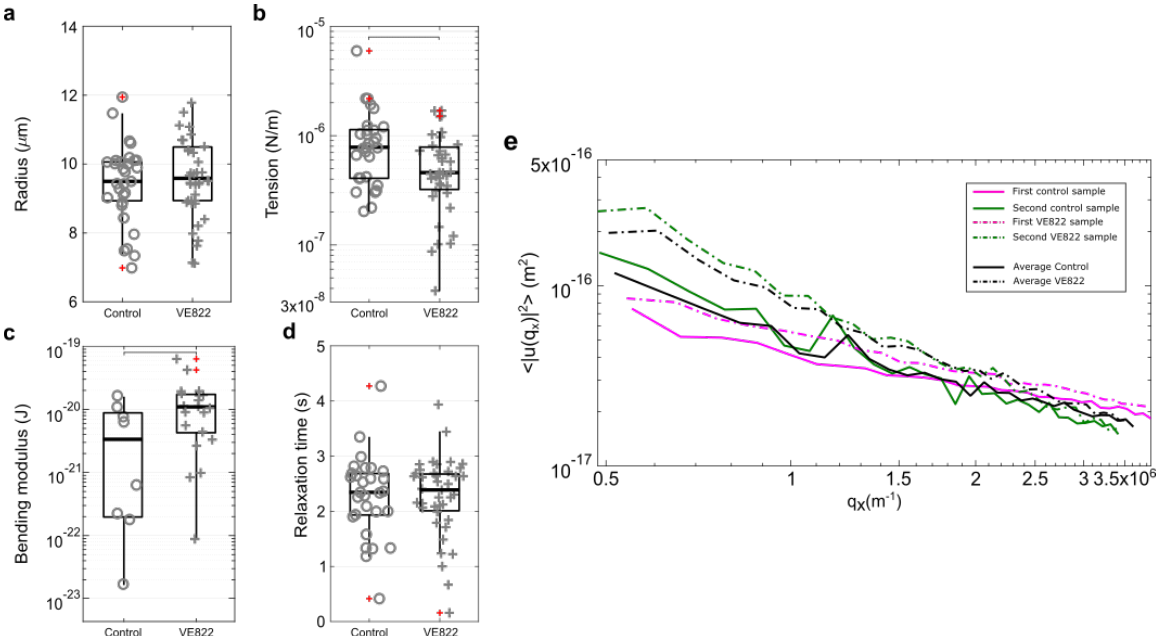


Effective tension is reduced (P value = 0.0218) and effective bending modulus increases (p value = 0.0338) in cells treated with VE822, while radius and relaxation time for mode 3 do not change (**a-d**). Panel **e** shows the fluctuation spectra from the 2 sets of experiments. The increase of bending modulus could be due to changes in the lipid composition as suggested by Kidiyoor *et al.* 2020 [31]. Data from 2 sets of experiments: 29 cells for control, 38 cells for VE822 treatment. Pairwise statistical comparisons were performed using the two-sided Mann-Whitney U test and the two-sample t-test after checking whether the data followed a Gaussian distribution.

**Figure S12.** Summary of nuclear contour fluctuations throughout the cell cycle and after chemical perturbations

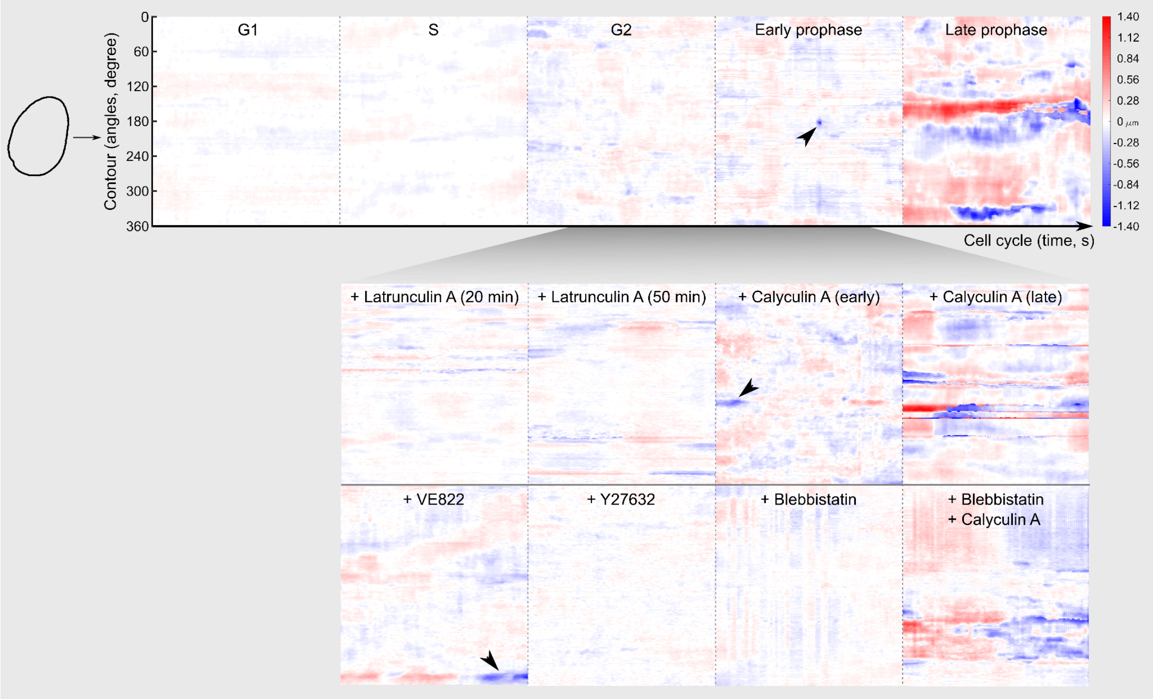


Heat maps highlighting the contour fluctuations of representative nuclei (see Videos) at different times of the cell cycle and after treatments. For each nucleus, the x axis indicates the duration of the recorded video (500 frames = 125 s), the y axis the contour profile angle, and the color map is the deviation from the mean contour (negative inward, positive outward). Black arrows indicate transient and localised invaginations that characterise early prophase and they are similarly visible after Calyculin A early treatment.

**SI Video S1.** Nuclear shape fluctuations in interphase (G1,S,G2) nuclei.

**SI Video S2.** Nuclear shape fluctuations in mitotic (early and late prophase) nuclei. NE labeled with Emerin-GFP and chromatin labeled with H2BmCherry. Scale bar =5µm

**SI Video S3.** Nuclear shape fluctuations in calyculin A treated (early and late) nuclei.

**SI Video S4.** Nuclear shape fluctuations in latrunculin A treated (early and late) nuclei.

**SI Video S5.** Nuclear shape fluctuations in blebbistatin and calyculin A treated nuclei.

**SI Video S6.** Correlation between the fluorescence signal of histones and NE deformation during transient invaginations

**SI Video S7.** Nucleus showing MTOC-derived irreversible invaginations preceding NEBD.

**SI Video S8.** Nuclear shape fluctuations of ATR inhibited-nuclei arrested in early prophase and treated with VE822.

**SI Video S9.** Representative 3D kymograph of nuclear shape fluctuations during late prophase

**SI Video S10.** Entire prophase of mitosis from chromatin condensation to nuclear envelope breakdown (total recording of 20 minutes at 5 seconds/frame rate).

**SI Video S11.** Nuclear shape fluctuations of ROCK inhibited-nuclei treated with Y27632.

**SI Video S12.** Nucleus showing local transient invaginations at the G2/M boundary.
