# Supplementary material for: Centripetal nuclear shape fluctuations associate with chromatin condensation in early prophase
Source: Commun Biol. 2023 Jul 12;6:715. doi: 10.1038/s42003-023-05074-9 (PMC10338514; doi:10.1038/s42003-023-05074-9)
Supplement: Supplementary file 3 — Description of Additional Supplementary Files [file 42003_2023_5074_MOESM3_ESM.pdf]

Description of Additional Supplementary Files

Video S1. Nuclear shape fluctuations in interphase (G1,S,G2) nuclei.

Video S2. Nuclear shape fluctuations in mitotic (early and late prophase) nuclei. NE labeled with Emerin-GFP and chromatin labeled with H2BmCherry. Scale bar =5µm

Video S3. Nuclear shape fluctuations in calyculin A treated (early and late) nuclei.

Video S4. Nuclear shape fluctuations in latrunculin A treated (early and late) nuclei.

Video S5. Nuclear shape fluctuations in blebbistatin and calyculin A treated nuclei.

Video S6. Correlation between the fluorescence signal of histones and NE deformation during transient invaginations

Video S7. Nucleus showing MTOC-derived irreversible invaginations preceding NEBD.

Video S8. Nuclear shape fluctuations of ATR inhibited-nuclei arrested in early prophase and treated with VE822.

Video S9. Representative 3D kymograph of nuclear shape fluctuations during late prophase

Video S10. Entire prophase of mitosis from chromatin condensation to nuclear envelope breakdown (total recording of 20 minutes at 5 seconds/frame rate).

Video S11. Nuclear shape fluctuations of ROCK inhibited-nuclei treated with Y27632.

Video S12. Nucleus showing local transient invaginations at the G2/M boundary.
